# Outcomes of Emergency Medical Service Usage in Severe Road Traffic Injury during Thai Holidays

**DOI:** 10.5811/westjem.2017.11.35169

**Published:** 2018-02-20

**Authors:** Sattha Riyapan, Phanthanee Thitichai, Wansiri Chaisirin, Tanyaporn Nakornchai, Tipa Chakorn

**Affiliations:** * Faculty of Medicine Siriraj Hospital, Mahidol University, Department of Emergency Medicine, Bangkok, Thailand; † Bureau of Epidemiology, Ministry of Public Health, Mueang Nonthaburi, Nonthaburi, Thailand

## Abstract

**Introduction:**

Thailand has the highest mortality from road traffic injury (RTI) in the world. There are usually higher incident rates of RTI in Thailand over long holidays such as New Year and Songkran. To our knowledge, there have been no studies that describe the impact of emergency medical service (EMS) utilization by RTI patients in Thailand. We sought to determine the outcomes of EMS utilization in severe RTIs during the holidays.

**Methods:**

We conducted a retrospective review study by using a nationwide registry that collected RTI data from all hospitals in Thailand during the New Year holidays in 2008–2015 and Songkran holidays in 2008–2014. A severe RTI patient was defined as one who was admitted, transferred to another hospital, or who died at the emergency department (ED) or during referral. We excluded patients who died at the scene, those who were not transported to the ED, and those who were discharged from the ED. Outcomes associated with EMS utilization were identified by using multiple logistic regression and adjusted by using factors related to injury severity.

**Results:**

Overall we included 100,905 patients in the final analysis; 39,761 severe RTI patients (39.40%; 95% confidence interval [CI] 95% CI [39.10%–39.71%]) used EMS transportation to hospitals. Severe RTI patients transported by EMS had a significantly higher mortality rate in the ED and during referral than that those who were not (2.00% vs. 0.78%, *p* < 0.001). Moreover, EMS use was significantly associated with increased mortality rate in the first 24 hours of admission to hospitals (1.38% for EMS use vs. 0.57% for no EMS use, *p* < 0.001). EMS utilization was a significant predictor of mortality in EDs and during referral (adjusted odds ratio [OR] 2.19; 95% CI [1.88–2.55]), and mortality in the first 24 hours of admission (adjusted OR 2.31; 95% CI [1.95–2.73]).

**Conclusion:**

In this cohort, severe RTI patients transported by EMS had a significantly higher mortality rate than those who went to hospitals using private vehicles during these holidays.

## INTRODUCTION

Road traffic injury (RTI) is a major public health issue. Every year, approximately 1.25 million people die and 20–50 million people worldwide are injured from RTI.^1^ RTI also has a huge economic impact, owing to costs of treatment, rehabilitation, accident investigation, and lost productivity. Thailand has the highest RTI mortality rate in the world,^1^ which may be attributed in part to the high incidence of drunk driving, high-speed driving, and low incidence of helmet or seatbelt use. The problem is further compounded by poor road conditions, especially in rural areas.^2^

The incidence of RTIs in Thailand increases during long holiday seasons – such as the New Year’s holiday December 31 – January 1 and the traditional Thai New Year’s holiday called Songkran, April 13 – 15 – because of high traffic volume and a higher incidence of drunk driving.^2^ The high-traffic holiday volume usually lasts seven days since many people add vacation leave to extend their time off.^2^ Since 2008 the country has collected RTI data during the holidays as part of a nationwide registry, which has been used for monitoring the incidence of RTIs, establishing public health interventions to prevent accidents, and improving post-crash response, including emergency medical services (EMS).

Similar to other countries, the Thai EMS system was developed as a part of the larger healthcare system, which aims to reduce morbidity and mortality in all emergencies. However, a recent study revealed that EMS use among RTI patients did not improve survival rates.^3^ These findings are similar to those of other studies focusing on EMS utilization in all patients with traumatic injuries.^4^–^6^ This indicates that improvement in prehospital care is needed, particularly for RTI patients. There is no global standard solution for RTI management. Understanding the characteristics of EMS utilization and its impact in individual regions is a critical component for improving the quality of the system. To our knowledge, no previous studies have described the impact of EMS utilization on road traffic accidents in Thailand.

As a first step toward improving the prehospital trauma care system in Thailand, which has a high incidence of RTIs, we sought to determine the outcomes of EMS utilization among RTI patients during the New Year and Songkran holidays, using data from a nationwide registry. Furthermore, the results may help other countries establish benchmarks to improve their EMS systems.

## METHODS

Thailand is a middle-income country in Southeast Asia. In 2015 its estimated population was 67,959,000, with a density of 132.1 per square kilometer.^7^ The country has 76 provinces grouped into 13 regional offices (ROs). The Thai Ministry of Public Health allocates funds to these ROs,^8^ which includes a budget for EMS organization. The Thai EMS system has been developing since 1995. It includes a two-tiered response, ambulance system that can be activated by dialing 1669. Basic life support (BLS) is provided by nonpublic health-sector organizations and hospital-based ambulances. BLS providers are basic emergency medical technicians (EMT-Bs) who have trained for at least 110 hours, or first responders who have trained for at least 40 hours.

The system provides advanced life support (ALS), administered by nurses through hospital-based ambulances. Certain rural areas have intermediate life support (ILS) provided by intermediate-level EMTs (EMT-I). EMT-Is complete a two-year training curriculum. In some areas that lack access to hospital-based ambulances, a first-response unit (FR) transports patients to hospitals. In 2008 the National Institute of Emergency Medicine (NIEM) was established to regulate EMS policies, EMS quality, and the licensure of EMS providers.^9^

**Population Health Research Capsule**

What do we already know about this issue?

Thailand has a high mortality rate from road traffic injury (RTI), especially over long holiday periods. There is little data describing the impact on the Thai EMS system from more RTI patients during the holidays.

What was the research question?

Was there a difference in outcomes of RTI patients transported by EMS compared to those who were transported by private vehicle?

What was the major finding of the study?

The EMS utilization group had a significantly higher severity and in-hospital mortality rate than the private vehicle group.

How does this improve population health?

The Thai trauma system should be improved to allow earlier access to and resuscitation of patients with RTI, especially those transported by EMS.

In an effort to prevent RTIs and control the quality of the EMS system during long holidays NIEM established a surveillance system in 2008 that collects RTI data from all hospitals. The surveillance collected data for seven days during each period from the Road Safety Directing Center’s announcements. For example, data were collected from December 27, 2013, to January 2, 2014, for the New Year’s 2014 holiday and from April 11 – 17 2014 for the Songkran holiday. NIEM used the registry to create prevention campaigns for the entire country, one example of which was the “Don’t Drive Drunk “campaign. NIEM also shared the data with police departments as a means to improve law enforcement in each province.

The RTI data-collection form included the name, sex, age, status of road user (e.g., driver, passenger, pedestrian), vehicle of patient, vehicle of party, date and time of accident, type of road (e.g., highway, rural, city) and the province in which the accident occurred, helmet/seatbelt use, alcohol use, admission/referral status, treatment outcome, length of stay, and post-crash transportation of patient. The form was distributed to all community, provincial, university, and private hospitals during the New Year and Songkran festival periods for the collection of data from all crash victims who accessed hospital care. Assigned data collectors interviewed patients or their relatives. Data on demographics, crash details, and risk factors were collected from interviewing patients or relatives or those who transported patients to the hospital. Data on treatment, outcome and post-crash transportation were collected from medical records. If a patient was admitted to the hospital, the outcome of treatment was reassessed at day 30 after a crash to be consistent with the World Health Organization definition of road traffic death. Alcohol-use information was obtained in various ways: from patients who verbally indicated that they had consumed alcohol prior to the injury; from relatives or from those who transported patients to the hospital; or by physical examination by a health provider, or laboratory testing. These data were then entered into the NIEM electronic database.

This study was reviewed and approved by the Faculty of Medicine Siriraj Hospital Institutional Review Board. We conducted a retrospective review study using data collected by the NIEM registry during the 2008–2015 New Year holiday and 2008–2014 Songkran holiday. We excluded patients who died at the scene and those who were not transported to hospitals because no transportation method for these patients had been recorded in the registry. Severe RTI patients were defined as patients who were admitted to the hospital, were referred, or had died in the emergency department (ED). We also excluded patients who were discharged from EDs.

Subsequently, we categorized data into two cohorts: a control group, and an EMS utilization group, which included patients who were transported to hospitals by FR, BLS, ILS, or ALS ambulances. The registry also recorded the vehicle type. We further classified the data according to whether the victims were vulnerable road users (which included pedestrians, cyclists, and motorcyclists) or non-vulnerable road users.^10^ We also categorized the time of the day that patients visited hospitals, dividing the day by 06:00–17:59 and 18:00–05:59. Patients who used helmets and seatbelts were combined. Mortality included death in EDs and during referral, death in the initial 24 hours after admission, and death 1–30 days after admission. Survival was defined as patients who either survived after 30 days of admission or were discharged from hospital.

We analyzed all demographic data comparing EMS and non-EMS utilization using chi-square test. Logistic regression was used to analyze the primary outcome, which was the association between EMS utilization and mortality of RTI patients; we then adjusted the outcome for factors that affected injury severity, such as age, sex, being a vulnerable road user, road characteristics, alcohol consumption, and helmet or seatbelt use.^11^–^18^ We conducted subgroup analyses to identify factors related to the mortality of patients who were transported by EMS. Univariate analysis was conducted using chi-square test and Fisher’s exact test. We included factors that have been proven to be associated with RTI severity, as mentioned above, and level of EMS in a multiple logistic regression model. *P* values <0.05 were considered significant. We calculated statistics using R version 3.2.1 (The R Foundation for Statistical Computing, Vienna, Austria).

## RESULTS

The nationwide registry of the New Year’s holiday reported 214,950 RTI patients in 2008–2015, and that of the Songkran holiday 202,298 RTI patients in 2008–2014. After excluding patients who died at the scene, were not transported to hospitals, or were discharged from the ED, 100,905 severe RTI patients were included in the final analysis (Figure). In total, 39,761 RTI patients (39.40%; 95% confidence interval [CI] 95% CI [39.10–39.71]) were transported by EMS.

Table 1 summarizes the demographic characteristics of severe RTI patients during these two major Thai holidays, categorized by mode of transportation. The mean (SD) age of patients transported by EMS was 32.12 (16.30) years, which was significantly greater than that of patients from the non-EMS utilization group (30.38 (17.19); *p* < 0.001). The severe RTI patients in this registry were predominantly male (73.86%). In the EMS utilization group 74.68% were male, and in the control group 73.26% were male. Approximately one-third of the accidents occurred on highway roads (31.53%), of which 38.08% of the victims were transported to the ED by ambulance, which was significantly more than those who were not transported (27.27%). Only 12,945 patients (13.98%) wore a helmet or seatbelt. Almost half of the severe RTI patients (50.88%) were influenced by alcohol. The history of alcohol consumption among patients transported by ambulance was significantly higher than in the control group (54.72% vs. 48.45%; *p* < 0.001).

The mortality rate in EDs and during referral in severe RTI patients transported by EMS was significantly higher than in those who were not (2.00% vs. 0.78%; *p* < 0.001). Moreover, EMS use revealed a mortality rate of 1.38% in the first 24 hours after admission to hospitals, which was significantly higher than the corresponding rate of 0.57% in the control group (*p* < 0.001). EMS utilization was a significant predictor of mortality in EDs and during referral, mortality in the first 24 hours of admission, and mortality in the 1–30 day period following admission to hospitals (Table 2). Following adjusted odds ratio (OR) with age, sex, RO, holiday year, helmet or seatbelt use, alcohol consumption, vulnerable road users, and road characteristics, EMS utilization had 2.19 times higher odds of mortality in EDs and during referral (adjusted OR 2.19; 95% CI [1.88–2.55]). It also significantly increased mortality in the first 24 hours after admission (adjusted OR 2.31; 95% CI [1.95–2.73]). Furthermore, EMS use increased the odds of mortality in the 1–30 day period following admission to the hospital (adjusted OR 1.57; 95% CI [1.28–1.92]).

Table 3 shows the characteristics of severe RTI patients transported by EMS, categorized by clinical outcomes. The patients who survived after 30 days of admission had been transported by ALS teams significantly less than patients who died (26.91% vs. 64.24% mortality in EDs and during referral, 58.18% mortality in the first 24 hours after admission, and 58.36% mortality in 1–30 days after admission to the hospital; *p* < 0.001). On the other hand, 14.42% of survival patients wore a helmet or seatbelt, which was significantly higher than among the patients who died (11.36% mortality in EDs and during referral, 9.47% mortality in first 24 hours after admission, and 9.21% mortality in 1 – 30 days after admission to the hospital; *p* < 0.001).

Multiple logistic regression revealed factors associated with mortality in EDs and during referral, mortality in the first 24 hours after admission, and mortality in 1 – 30 days after admission to the hospital, (Tables 4, 5, and 6, respectively). ALS transportation was a significant factor in increased mortality (OR 4.63; 95% CI [3.72–5.82] in mortality in EDs and during referral, OR 3.44; 95% CI [2.73–4.35] in mortality in the first 24 hours after admission, and OR 3.61; 95% CI [2.65–4.96] mortality in 1 – 30 days after admission). Accidents on highway roads increased the odds of mortality in EDs and during referral than those on city roads (OR 1.57; 95% CI [1.19–2.11]). Highway-related injuries also had 1.52-times higher odds of mortality in the first 24 hours of admission (OR 1.52; 95% CI [1.11–2.11]). In contrast, helmet or seatbelt use was a significant factor in decreasing mortality rates (OR 0.56; 95% CI [0.41–0.76] in ED mortalities and during referral, OR 0.64; 95% CI [0.45–0.90] in mortality in the first 24 hours after admission, and OR 0.52; 95% CI [0.30–0.84] mortality in 1 – 30 days after admission).

## DISCUSSION

This study describes outcomes of severe RTI patients transported by EMS compared with patients transported by private vehicles. We conducted the analysis using a nationwide registry in Thailand, which has the highest traffic accident mortality rate in the world. Moreover, the registry collected data during holidays with a high incidence of RTIs. In this cohort, severe RTI patients transported by ambulance had a higher mortality rate than patients transported to hospitals by private vehicles, and this finding is in line with those of other studies.^3^,^4^,^19^ The higher mortality rate might be attributed to the fact that patients who were transported by EMS were more severely injured than those in the control group.

Our results demonstrated that approximately 40% of severe RTI patients were transported to hospitals by ambulance, which was less than reports from other countries. Recently, Huang et al. reported that 73.4% of RTI patients in Taiwan were transported by EMS. One possible reason for not using EMS may have been that patients might not have known or might have forgotten the four-digit (1669) number for ambulance services.^20^ This contact number is different from those of other public service agencies such as the police and fire departments. A continuous advertisement of the emergency number should be done to increase EMS use in Thailand.

The demographic data revealed that the patients who used EMS had more factors that increased injury severity than the control group; for example, our analysis demonstrated that severe RTI patients transported by ambulance reported current alcohol consumption more than those who were not. Recent studies have reported that alcohol intoxication is associated with greater injury severity and higher mortality rates among RTI patients.^17^,^21^–^23^ We also found that severe RTI patients transported by EMS were more often injured on highways than patients who were not. This shows that the patients in the EMS use group were more severely injured than those in the control group, since accidents on highways were more likely to have occurred at high speed, which was associated with more severe injuries.^14^ Although we analyzed multiple logistic regression to adjust for confounding factors, certain factors related to injury severity were not included in the registry, such as vehicle speed, comorbidity, prehospital care time, or injury severity scores (ISS).^14^,^24^ To find out the real effect of EMS use in clinical outcomes of RTI patients, the registry should collect information about other factors related to severity of injuries.

The subgroup analysis identified factors associated with mortality among severe RTI patients transported by EMS. It demonstrated that patients who were transported by ALS teams had significantly increased mortality. This might have been due to the fact that patients transported by ALS teams were at higher risk of increased severity when compared with patients transported by other EMS levels. Another possibility is that ALS team use might increase the on-scene time, due to prehospital interventions such as administering intravenous fluids or performing endotracheal intubation, as opposed to the “scoop and run” concept. This concurs with findings of previous studies that the use of ALS teams did not improve clinical outcomes.^25^,^26^

The Ontario Prehospital Advanced Life Support Major Trauma Study demonstrated that implementation of ALS teams did not improve the survival of major trauma patients when compared with patients treated by BLS teams.^26^ The study also revealed that among patients with a Glasgow Coma Score less than 9 who needed endotracheal intubation, those transported by ALS teams had a significantly lower survival rate.^26^ However, our registry did not collect prehospital care time and prehospital intervention. Further studies should be conducted to determine the real effect of ALS teams on the clinical outcomes of RTI patients by analyzing all confounding factors.

Helmet or seatbelt use was a factor that reduced mortality in severe RTI patients transported by ambulance. This concurred with the findings of previous studies that showed that these protective devices reduce injury severity.^13^,^27^,^28^ Abu-Zidan et al. reported that restrained RTI patients showed significantly less severe injury as well as fewer surgeries compared with unrestrained patients.^27^ Furthermore, Nash et al. reported that seatbelt use was associated with a significant reduction in injury severity, mortality rate, and length of stay among RTI patients.^28^ Liu et al. reviewed 61 observational studies and found that helmet use was a significant factor in reducing mortality and head injury in motorcycle crashes.^13^ Only 13.89% of patients in this cohort wore a helmet or seatbelt, although Thai law requires helmet and seatbelt use. Further studies should be conducted to explore barriers to helmet and seatbelt use.

## LIMITATIONS

Although we analyzed data from the largest RTI registry in the country, revealing high mortality rates among RTI patients, our study has certain limitations. First, because it was a retrospective observational study, there were missing data regarding accident time, helmet and seatbelt use, alcohol consumption status, and road characteristics. Second, the collection method in the registry had a potential for recall bias since the data collectors interviewed patients or their relatives at the hospital. Third, the registry collects data only in the long holiday periods. Given the lack of data for non-holidays the registry could not represent the effect of EMS utilization during non-holidays. We also excluded patients who died at the scene and were not transported to hospitals. This might have changed the injury severity of the whole population.

Furthermore, the analysis combined patients using helmet and seatbelt in one variable as a protective factor for overall RTI patients, when the injuries have different mechanisms. Further investigation should conduct subgroup analysis comparing those using motorcycles vs. four-wheel vehicles. Moreover, as mentioned earlier, the registry did not collect data on confounding variables that could affect clinical outcomes, such as ISS, prehospital care time, vehicle speed, or patient comorbidities. Lacking prehospital intervention is another limitation. The registry should collect the prehospital management or link with the Thai EMS database. Alcohol consumption was defined using patient history data and physical examination by the physician, which is not the gold standard. Blood alcohol levels should perhaps be included in the registry. Improving the registry will help enhance our understanding of these characteristics as well as the effects of EMS utilization on clinical outcomes.

Aside from our suggestions to improve the registry, there are further implications from the results of this study. Since we found that the RTI patients transported by EMS during the holidays had increased mortality rates, we recommend that this group of patients be evaluated in a trauma resuscitation room earlier, especially for patients transported by ALS teams. And to reduce time spent in the ED, prehospital notification should be given to receiving hospitals by the ambulance team before arrival.

## CONCLUSION

Transportation of severe road traffic injury patients by EMS was significantly associated with increased mortality in EDs and during referral, as well as mortality in the first 24 hours following hospital admission and mortality from 1 – 30 days after admission. However, certain additional confounding factors must be collected for adjustment in these associations. We recommend improving the Thai RTI registry by reducing confounders. This will enable researchers to identify the actual effects of EMS utilization among severe RTI patients in Thailand. Furthermore, we suggest that severe RTI patients be taken to hospitals during these holidays by ambulance and, especially those taken by ALS team, should be rapidly assessed in the ED. These changes could potentially improve the clinical outcomes of RTI patients in Thailand.

## Figures and Tables

**Figure:**
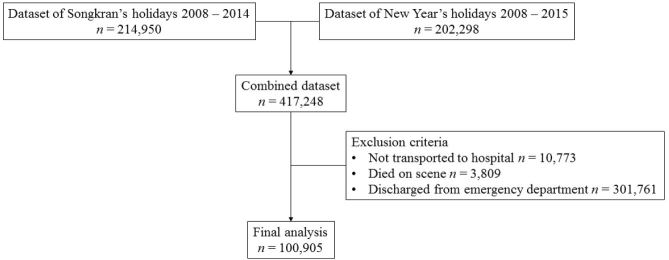
Flow chart of dataset for analysis of road traffic injuries in Thailand during Songkran’s and New Year’s holidays.

**Table 1:** Demographic data of severe road traffic injury patients categorized by EMS* transportation.

Variable	Overall n = 100,905	Non-EMS utilization n = 61,144	EMS Utilization n = 39,761
Age group			
0–8 years old	5,370 (5.32%)	4,090 (6.69%)	1,280 (3.22%)
9 – 17 years old	17,147 (16.99%)	10,989 (17.97%)	6,158 (15.49%)
18 – 60 years old	72,320 (71.67%)	42,418 (69.37%)	29,902 (75.20%)
61 years old and more	6,068 (6.01%)	3,647 (5.96%)	2,421 (6.09%)
Male	74,529 (73.86%)	44,796 (73.26%)	29,733 (74.78%)
Vulnerable road users	85,052 (84.29%)	52,545 (85.94%)	32,507 (81.76%)
Night shift (1800 – 0599) (Missing n = 746)	47,544 (47.47%)	28,348 (46.76%)	19,196 (48.56%)
Road type (Missing n = 2,443)			
City road	15,781 (15.64%)	9,405 (15.38%)	6,376 (16.04%)
Rural road	50,867 (50.41%)	33,361 (54.56%)	17,506 (44.03%)
Highway road	31,814 (31.53%)	16,672 (27.27%)	15,142 (38.08%)
Helmet or seatbelt (Missing n = 7,700)	12,945 (13.89%)	7,733 (13.64%)	5,212 (14.27%)
Alcohol consumption (Missing n = 7,523)	47,517 (50.88%)	27,657 (48.45%)	19,860 (54.72%)
Injury in Songkran holiday	47,468 (47.04%)	29,507 (48.26%)	17,961 (45.17%)
Outcomes			
Survival after 30 days	98,202 (97.32%)	60,069 (98.24%)	38,133 (95.90%)
Death in ED and referral	1,271 (1.26%)	474 (0.78%)	797 (2.00%)
Death in first 24 hours of admission	897 (0.89%)	347 (0.57%)	550 (1.38%)
Death in 1 day – 30 days after admission	535 (0.53%)	254 (0.42%)	281 (0.71%)

*EMS*, emergency medical services; *ED*, emergency department

**Table 2:** Multiple logistic regression for emergency medical service utilization among severe road traffic injury patients and mortality

Outcomes	Crude OR (95%CI)	Adjusted OR* (95%CI)
Mortality in EDs and during referral	2.62 (2.37 – 2.94)	2.19 (1.88 – 2.55)
Mortality in first 24 hours of admission to hospitals	2.46 (2.15 – 2.81)	2.31 (1.95 – 2.73)
Mortality in 1 – 30 days after admission to hospitals	1.71 (1.44 – 2.02)	1.57 (1.28 – 1.92)

*OR*, odds ratio; *CI*, confidence interval; *ED*, emergency department.

* Adjusted with factors that affected injury severity such as age, sex, vulnerable road users, road characteristics, alcohol consumption, and helmet or seatbelt usage.^11^–^18^

**Table 3:** EMS* transport of patients with severe road traffic injuries, classified by clinical outcomes

Variable	Survival after 30 days n = 38,133	Death in EDs and during referral n = 797	Death in first 24 hours after admission n = 550	Death in 1 day – 30 days after admission n = 281	P value
Age					<0.001
0–8 years old	1242 (3.27%)	22 (2.76%)	12 (2.18%)	4 (1.42%)	
9 – 17 years old	5971 (15.66%)	75 (9.41%)	82 (14.91%)	30 (10.68%)	
18 – 60 years old	28668 (75.18%)	615 (77.16%)	404 (73.45%)	215 (76.51%)	
>61 years old	2252 (5.91%)	85 (10.66%)	52 (9.45%)	32 (11.39%)	
Male	28479 (74.68%)	605 (75.91%)	434 (78.91%)	215 (76.51%)	0.105
Vulnerable Road Users	31261 (81.98%)	578 (72.52%)	437 (79.45%)	231 (82.21%)	<0.001
Night shift* (Missing n = 231)	18368 (48.45%)	397 (50.00%)	294 (53.75%)	137 (48.75%)	0.081
Road type (Missing n =737)					<0.001
City road	6143 (16.11%)	107 (13.43%)	80 (14.55%)	46 (16.37%)	
Rural road	16950 (44.45%)	237 (29.74%)	208 (37.82%)	111 (39.50%)	
Highway road	14328 (37.57%)	441 (55.33%)	256 (46.55%)	117 (41.63%)	
Helmet or seatbelt (Missing n = 3,246)	5077 (14.42%)	70 (11.36%)	43 (9.47%)	22 (9.21%)	<0.001
Alcohol consumption (Missing n = 3,464)	38133 (54.74%)	232 (48.03%)	228 (57.43%)	129 (60.56%)	0.005
Injury in Songkran holiday Holiday	17220 (45.16%)	359 (45.04%)	265 (48.18%)	117 (41.64%)	0.329
EMS level					<0.001
ALS	10261 (26.91%)	512 (64.24%)	320 (58.18%)	164 (58.36%)	
ILS	466 (1.22%)	6 (0.75%)	4 (0.73%)	1 (0.36%)	
BLS	7770 (20.38%)	85 (10.66%)	64 (11.64%)	31 (11.03%)	
FR	19636 (51.49%)	194 (24.34%)	162 (29.45%)	85 (30.25%)	

*ED*, emergency department;

* *EMS*, emergency medical services, *ALS* Advanced Life Support team; *ILS*, Intermediate Life Support team; *BLS*, Basic Life support team; *FR*, first responder.

* Night shift hours: 1800 – 0559

**Table 4:** Factors associated with mortality in the emergency department and during referral among severe RTI patients transported by EMS*.

Variables	Odds ratio (OR)	95% Confidence interval	P value
Male	1.09	0.86 – 1.39	0.465
Age			
0 – 8 years old	Reference		
9 – 17 years –old	0.62	0.35 – 1.13	0.099
18 – 60 years old	0.98	0.60 – 1.71	0.936
> 61 years old	1.53	0.87 – 2.82	0.154
Vulnerable road user	0.90	0.71 – 1.15	0.394
EMS levels			
FR	Reference		
BLS	1.16	0.82 – 1.61	0.387
ILS	1.51	0.46 – 3.61	0.423
ALS	4.63	3.72 – 5.82	<0.001
Road locations			
City roads	Reference		
Rural roads	0.84	0.62 – 1.15	0.263
Highway roads	1.57	1.19 – 2.11	0.002
Helmet or seatbelt usage	0.56	0.41 – 0.76	<0.001
Alcohol consumption	0.76	0.61 – 0.96	0.019

C=0.734

*ED*, emergency department;

* *EMS*, emergency medical services, *ALS* Advanced Life Support team; ILS, Intermediate Life Support team; *BLS*, Basic Life support team; *FR*, first responder; *RTI*, road traffic injury.

**Table 5:** Factors associated with mortality in the first 24 hours of hospital admission among severe RTI patients transported by EMS*

Variables	Odd ratio (OR)	95% Confidence interval	P value
Male	1.13	0.86 – 1.50	0.383
Age			
0 – 8 years old	Reference		
9 – 17 years –old	1.47	0.71 – 3.57	0.340
18 – 60 years old	1.52	0.76 – 3.58	0.284
> 61 years old	3.19	1.52 – 7.80	0.005
Vulnerable road user	1.06	0.80 – 1.44	0.679
EMS levels			
FR	Reference		
BLS	1.00	0.70 – 1.41	0.998
ILS	1.09	0.27 – 2.91	0.880
ALS	3.44	2.73 – 4.35	<0.001
Road locations			
City roads	Reference		
Rural roads	1.03	0.75 – 1.44	0.865
Highway roads	1.52	1.11 – 2.11	0.011
Helmet or seatbelt usage	0.64	0.45 – 0.90	0.013
Alcohol consumption	1.11	0.86 – 1.42	0.428

C=0.6996

*ED*, emergency department;,

* *EMS*, emergency medical services, *ALS* Advanced Life Support team; ILS, Intermediate Life Support team; *BLS*, Basic Life support team; *FR*, first responder; *RTI*, road traffic injury.

**Table 6:** Factors associated with mortality 1 to 30 days after hospital admission among severe RTI patients transported by EMS*

Variables	Odds ratio (OR)	95% Confidence interval	P value
Male	0.90	0.63 – 1.30	0.561
Age			
0 – 8 years old	Reference		
9 – 17 years –old	1.26	0.43 – 5.38	0.710
18 – 60 years old	2.14	0.80 – 8.71	0.196
> 61 years old	3.46	1.17 – 14.77	0.047
Vulnerable road user	0.97	0.67 – 1.46	0.887
EMS levels			
FR	Reference		
BLS	0.97	0.60 – 1.55	0.916
ILS	0.65	0.04 – 2.94	0.665
ALS	3.61	2.65 – 4.96	<0.001
Road locations			
City roads	Reference		
Rural roads	0.70	0.48 – 1.04	0.071
Highway roads	0.92	0.63 – 1.36	0.656
Helmet or seatbelt usage	0.52	0.30 – 0.84	0.013
Alcohol consumption	1.28	0.91 – 1.81	0.157

C=0.708

*ED*, emergency department;

* *EMS*, emergency medical services, *ALS* Advanced Life Support team; *ILS*, Intermediate Life Support team; *BLS*, Basic Life support team; *FR*, first responder; *RTI*, road traffic injury.
